# Heterogeneity and changes in preferences for dying at home: a systematic review

**DOI:** 10.1186/1472-684X-12-7

**Published:** 2013-02-15

**Authors:** Barbara Gomes, Natalia Calanzani, Marjolein Gysels, Sue Hall, Irene J Higginson

**Affiliations:** 1 King’s College London, Cicely Saunders Institute, Department of Palliative Care, Policy & Rehabilitation, Bessemer Road, London, SE5 9PJ, UK

**Keywords:** Patient preference, Hospice care, Palliative care, Hospices, Terminal care, Patient satisfaction

## Abstract

**Background:**

Home-based models of hospice and palliative care are promoted with the argument that most people prefer to die at home. We examined the heterogeneity in preferences for home death and explored, for the first time, changes of preference with illness progression.

**Methods:**

We searched for studies on adult preferences for place of care at the end of life or place of death in MEDLINE (1966–2011), EMBASE (1980–2011), psycINFO (1967–2011), CINAHL (1982–2011), six palliative care journals (2006–11) and reference lists. Standard criteria were used to grade study quality and evidence strength. Scatter plots showed the percentage preferring home death amongst patients, lay caregivers and general public, by study quality, year, weighted by sample size.

**Results:**

210 studies reported preferences of just over 100,000 people from 33 countries, including 34,021 patients, 19,514 caregivers and 29,926 general public members. 68% of studies with quantitative data were of low quality; only 76 provided the question used to elicit preferences. There was moderate evidence that most people prefer a home death–this was found in 75% of studies, 9/14 of those of high quality. Amongst the latter and excluding outliers, home preference estimates ranged 31% to 87% for patients (9 studies), 25% to 64% for caregivers (5 studies), 49% to 70% for the public (4 studies). 20% of 1395 patients in 10 studies (2 of high quality) changed their preference, but statistical significance was untested.

**Conclusions:**

Controlling for methodological weaknesses, we found evidence that most people prefer to die at home. Around four fifths of patients did not change preference as their illness progressed. This supports focusing on home-based care for patients with advanced illness yet urges policy-makers to secure hospice and palliative care elsewhere for those who think differently or change their mind. Research must be clear on how preferences are elicited. There is an urgent need for studies examining change of preferences towards death.

## Background

Debates about the institutionalization of death and the fact that most people die in hospital have happened within and between countries since the beginning of the hospice movement [1-3]. The issue has gained momentum in light of national and international projections showing dramatic rises in numbers of deaths due to increased life expectancy and large cohorts of “baby-boomers” reaching older ages [4-6]. As chronic conditions are increasingly leading causes of death [4,7], most people should expect a period of terminal illness to precede death. This urged projections of place of death, with a view to inform how the care of terminally ill patients is planned for the future [8-10].

Many countries including the United States (US) and the United Kingdom (UK) have increased the focus on home-based models of hospice and palliative care with the argument that most people prefer to die at home. Trends of increasing home deaths have followed [11-14]. Indeed, a review of the cancer literature in 2000 showed that more than 50% of patients, lay caregivers and members of the public preferred to be cared for and to die at home [15]. However, the quality of the 18 studies examined was low and the percentage of people who preferred home varied greatly, from 25% to 100%. A methodological review in 2009 highlighted differences in methods of eliciting and reporting preferences [16], but there may be other reasons why a preference for home is high in some studies and low in others. It is commonly accepted that dying in hospital remains frequent because people change their mind as their illness progresses, based in two studies conducted in the 1980s in London, UK [17,18].

We aimed to examine the heterogeneity in estimates of a preference for home death. We explored reasons for variation, particularly in relation to the quality of studies, the way in which preferences were assessed and, for the first time, examined changes of preference with illness progression.

## Methods

### Design

Systematic review. The PRISMA checklist is available online in Additional file 1.

### Search strategy

Three strategies were used to systematically identify relevant studies. First, in May 2011 we searched four databases - MEDLINE (1966–2011), EMBASE (1980–2011), psycINFO (1967–2011) and CINAHL (1982–2011) using a combination of MESH headings and 22 keywords (details online in Additional file 2). Secondly, we handsearched six palliative care journals January 2006 – June 2011 (*Palliative Medicine, Journal of Palliative Medicine, Supportive Care in Cancer, Journal of Pain and Symptom Management, BMC Palliative Care, Journal of Palliative Care*) to identify recent articles not available through databases. Thirdly, the references of 18 literature reviews and all included papers were tracked (references available from reviewers). Additional data on included studies were obtained via personal contact.

### Selection criteria

Inclusion criteria were: original data on people’s preferences for place of end of life care and/or place of death; a scenario (real or hypothetical) of advanced or severe stages of progressive disease (cancer or non-malignant); adult population; use of quantitative and/or qualitative methodologies (qualitative data aimed to complement areas with low or inconclusive quantitative evidence).

Exclusion criteria were: no examination of preferences but only factual place of care or place of death; preferences not explicitly for place of care at the end of life or place of death; preferences not framed in a scenario of advanced or severe stages of a progressive disease; children only; reviews, comments, case stories, historical, ethical or educational analysis or unpublished material; papers not written in English, French, German, Italian, Portuguese or Spanish (due to translation limits).

### Data extraction

The data were extracted to a standard form and SPSS datasheets under the following headings: *study type* (quantitative, qualitative or using both methods); *study design* (retrospective, cross-sectional, prospective – depending on the assessment timing of preferences); *population to which preferences referred to (*general public, patients, lay caregivers, older people, health care professionals, medical or nursing students); *country of origin; publication year; data collection year(s); setting* (including whether the study was population-based or service-based)*; sample* (including sample size, % with cancer and % in terminal stage for patient populations, i.e. with advanced/severe illness or who died before or during the study period); *response rate; preferences assessment method* (including which questions were asked and whether preferences referred to place of care or place of death)*; results* (including percentage expressing a preference for home, changes over time and qualitative findings)*.* BG or NC extracted the data from the papers and a second reviewer assessed a 10% sample of papers to check accuracy. Disagreements were resolved by consensus.

### Assessment of quality and strength of evidence

The quality of individual studies was assessed using two different standardized scales for quantitative and qualitative research ([19,20], details in Additional file 3). Studies using both quantitative and qualitative methods were evaluated using both scales. Studies were classified as high, medium or low quality using final scores (high ≥70%, medium 60%-69%, low <60%). However, because final quality scores for quantitative and qualitative research reflect different criteria, direct comparisons between the two are not appropriate. BG or NC assessed the quality of studies; SH independently assessed a 7% sample of studies using quantitative methods and MG assessed a 50% sample of studies using qualitative methods (we checked a higher percentage of qualitative studies as initial comparisons showed more disagreement). Disagreements were resolved by consensus.

We graded the strength of the evidence adapting an algorithm from a review on factors associated with death at home for cancer patients [21]. This made use of the key elements for grading systems recommended by the US Agency for Healthcare Research and Quality – quality, quantity and consistency of the evidence [22]. We took high strength evidence from a minimum of three high quality studies in which ≥70% reported similar findings. Moderate strength evidence was measured amongst medium and high quality studies and it was present if there was a minimum of three high quality studies in which <70% but >50% reported similar findings or if >50% of all studies reported similar findings with a minimum of three medium quality studies. If none of these requirements were met, the evidence was considered of low strength. The evidence was found inconclusive if the consistency was 50% (e.g. two studies showing different results) or when there was only one study. We graded the strength of evidence from all studies and of sub-groups: studies prior to 2000 and since 2000, studies in each population group, and studies examining changes of preference over time.

### Data synthesis

We described the included studies in terms of country of origin, populations, design, assessment of preferences (outlining which questions were asked, when available) and year of publication (prior to and since 2000). We used charts to summarize the results of the quality assessment, separately for quantitative and qualitative research. Percentages of participants expressing a preference for dying at home in each study were calculated or extracted from the papers if numbers were not given. Studies that did not provide enough information to extract or calculate the percentage of participants with a preference for dying at home were excluded from further quantitative synthesis. Heterogeneity precluded the calculation of a summary measure. Instead, we examined ranges in percentages and numbers of studies where >50% and >70% of the participants expressed a home preference. These were analyzed by population group and the strength of the evidence was determined. We tabulated the methods and results of high quality studies.

Percentages of participants with a home preference were plotted separately for members of the general public, patients and caregivers (groups with more than ten studies), and according to study quality (high, medium, low). Percentages were weighted by sample size and ordered by year of publication to identify patterns. In studies measuring preferences for both place of care and place of death, we plotted place of death preferences; these were deemed most relevant given the policy and trends of increasing home deaths. For longitudinal studies with multiple measurements of preferences we plotted the average. When ideal and realistic preferences were assessed and reported we plotted the realistic preferences only. Cases with missing data were excluded from calculations.

For studies conducted with patients, we distinguished those where the majority had cancer. Country of origin, whether the question referred to place of care or place of death, study quality and the questions used to assess preferences were explored as reasons for heterogeneity. A narrative summary of the findings in each of the three population groups is provided, discussing outlying studies, defined as those with extreme estimates and small samples (<100). We then analyzed changes in preferences over time, grading the strength of the evidence. We examined qualitative research on areas with low or inconclusive quantitative evidence, and integrated a narrative summary of the findings in the respective results section. Given the large number of studies included, we reference in the text only high quality studies included in the main analysis, outliers and key studies but all references are available from the reviewers.

## Results

### Searches, data extraction and disagreement between reviewers

**Figure 1:**
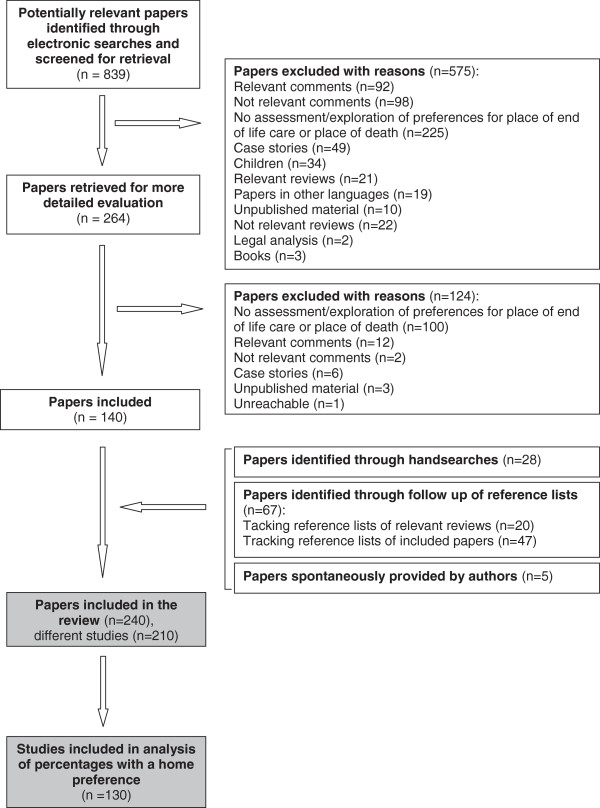
Flow of studies through review.

Electronic searches yielded 839 references (PRISMA flowchart in Figure 1). Through scrutiny of abstracts, almost a third appeared eligible for inclusion; after examination of full versions, 140 were included. Handsearches, tracking of reference lists and spontaneous provision of studies by the authors added 100 papers. A total of 240 papers were reviewed, reporting 210 different studies: three papers presented two different datasets in which preferences were assessed differently in two populations (considered different studies), one presented four different datasets, and 36 reported on data from already included studies (these were merged with the first report). There were disagreements between the reviewers on the quality classification of 12 studies (three on quantitative methods and nine on qualitative methods).

### General overview

The studies reported on the preferences for place of end of life care and/or place of death of 29,926 members of the general public, 34,021 patients, 19,514 caregivers, 11,613 older people, 3,504 health professionals (for their patients or themselves) and 1,729 medical or nursing students, from 33 countries.

Twenty-eight percent of the studies (n = 59) presented evidence from the US and 41% (n = 86) were conducted in 17 countries in Europe (Belgium, Denmark, England, France, Germany, Hungary, Ireland, Israel, Italy, Netherlands, Norway, Scotland, Spain, Sweden, Switzerland, Turkey and Wales). Other countries included Australia, Canada, Hong Kong, Japan, New Zealand, Singapore, South Korea, Taiwan, and seven African countries (Ethiopia, Ghana, Rwanda, South Africa, Tanzania, Uganda and Zimbabwe). One qualitative study (ethnographic) explored preferences for place of death in the US and Japan, and another qualitative study examined the preferences of young adults with cancer in the UK, Germany and Australia (based on written narratives from their parents); an online survey from the *British Medical Journal* was UK-based but had worldwide coverage.

Nearly three quarters of the studies (n = 151) focused on patients or caregivers. In 73 studies most patients had cancer whilst in 37 studies most had non-malignant diseases such as dementia, human immunodeficiency virus (HIV)/acquired immunodeficiency syndrome (AIDS), heart failure, chronic obstructive pulmonary disease (COPD) and motor neurone disease (MND). In four studies there were equal proportions of patients with cancer and non-malignant conditions, and in the remaining 37 the percentage with cancer was unknown. The patients were deemed terminally ill in 129 studies.

### Progression of studies, methodological quality and assessment of preferences

Only 34 out of the 210 studies were of high quality (16%), 51 were medium quality (24%) and 125 were low quality studies (60%). Four of the 49 studies published prior to 2000 were considered of high quality. Since 2000, 161 new studies were published, 30 of high quality (19%). Nearly three quarters of all studies used quantitative methods (n = 153), 41 used qualitative methods and 16 made use of both (the qualitative component was dominant in four of these).

**Figure 2:**
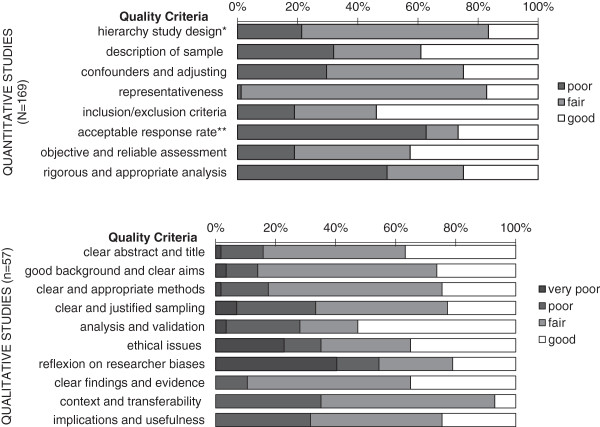
**Quality assessment for studies using quantitative and qualitative methods.** Footnote: Quantitative criteria applied to 169 studies (153 purely quantitative, 16 using both quantitative and qualitative methods). Qualitative criteria applied to 57 studies (41 purely qualitative and 16 using both quantitative and qualitative methods). * Rating for study design (poor = retrospective, fair = cross-sectional, good = prospective). ** Rating for acceptable response rate (poor <60% or unknown, fair 60%-69%, good ≥70%).

Around two thirds of the quantitative research was of low quality (n = 114/169). Fourteen studies used quantitative methods at high standard. The inclusion/exclusion criteria for participants were generally clear and the assessment of preferences objective and reliable (Figure 2). Main weaknesses were poor response rates, inadequate design, potential for confounding, and limited analysis. Most were cross-sectional, assessing preferences at one point in time, 36 did this retrospectively and 15 studies provided data on preferences over time. Response rates were either below 60% or unknown in around three quarters of the studies (n = 124).

More than a third (39%) of the qualitative research was considered of high quality (n = 22/57). Almost all studies had a good background, using appropriate and specific qualitative methods and sampling techniques with clear aims. Lack of reflection on researcher biases, validation in the analysis and ethical issues were weaker points. The samples were often small (27 studies had <40 participants), which limited the transferability of the findings (found to be good in four studies only).

Seventy-six studies provided the exact question used for assessing preferences (details in Additional file 4). The questions were varied, with some enquiring directly if home was the preferred place, whilst others asked more broadly what the preferred place was or would be. In addition, 31 studies did not state the question but either described it briefly or referred to the tool from where the question was taken, and 103 were not fully clear on the terminology/context used, or did not mention the question or tool used at all.

### Preference for dying at home

The percentage of participants who expressed a preference for dying at home was reported in 130 studies; 95 were published since 2000. Most examined preferences for place of death (n = 76), 41 referred to place of care and ten to both place of care and place of death (three were unclear). Only 14 studies were of high quality, all but three published since 2000 (available online in Additional file 5). The 14 high quality studies report on preferences for 6,463 people (1,400 patients, 836 caregivers and 4,227 members of the general public) (Table 1).

**Table 1:** Preferences for dying at home: quantity, quality, consistency and strength

**Population group and studies**		**High strength evidence (only high quality studies)**				**Moderate evidence (high and medium quality studies)**	
**Population group**	**Number of studies**	**Number of participants**	**Consistency**^**a**^	**Preference for dying at home**	**High strength?**	**Preference for dying at home**	**Moderate strength?**
**Evidence for >50% preference for dying at home**							
All people	130	6463	64% (9/14)	>50%	no	>50%	yes
Patients	92	1400	60% (6/10)	>50%	no	>50%	yes
Caregivers	36	836	60% (3/5)	>50%	no	>50%	yes
General public	26	4227	75% (3/4)	>50%	yes	n/a	n/a
Health professionals and students	7	0	0% (0/0)	>50%	no	>50%	yes
Older people	9	0	0% (0/0)	>50%	no	>50%	no
**Evidence for >70% preferences for dying at home (or <70%)**							
All people	130	6463	29% (4/14)	>70%	no	<70%	no
Patients	92	1400	50% (5/10)	>70%	no	<70%	no
Caregivers	36	836	80% (4/5)	<70%	yes	n/a	n/a
General public	26	4227	75% (3/4)	<70%	yes	n/a	n/a
Health professionals and students	7	0	0% (0/0)	>70%	no	>70%	yes
Older people	9	0	0% (0/0)	>70%	no	>70%	no

Footnote: The table shows the total number of studies that reported a >50% and a >70% (or <70%) preference for dying at home amongst all types of participants and within each of five population groups. It then shows whether there is high strength evidence amongst all people and within each population group. High strength evidence was measured amongst only high quality studies and it was present, according to our grading system, if ≥70% of studies reported similar findings (e.g. there was no high strength evidence that >50% of all people preferred dying at home because the consistency was 64%, i.e. nine out of 14 studies showed estimates higher than 50% but five did not). The last two columns show whether there is moderate strength evidence; this was measured amongst medium and high quality studies and it was present, according to our grading system, if there was a minimum of three high quality studies in which <70% but >50% reported similar findings or if >50% of all studies reported similar findings with a minimum of three medium quality studies (e.g. there was moderate strength evidence that >50% of all people preferred dying at home because the consistency amongst high quality studies was 73%, i.e. more than half of the studies showed estimates higher than 50%).

^a^Consistency of findings across studies is shown as a percentage (number of high quality studies pointing in same direction / total number of high quality studies on topic).

We found moderate strength evidence that the majority of people preferred dying at home (Table 1). In 75% of the studies (97/130) >50% of participants expressed a preference for home (ranging from 51% to 100%). However, the consistency of findings amongst high quality studies was below 70% (9/14), hence the evidence did not reach high strength. In around one third of all studies (47/130) the percentage of participants expressing a preference for home was higher than 70%.

Two high quality studies published prior to 2000 found that most participants preferred home – 59% amongst 462 members of the general public in Adelaide and three rural areas in Australia and 73% amongst 120 hospitalized AIDS patients in five Seattle tertiary care hospitals in the US [23,24]. Another high quality study, a longitudinal study of 77 home care patients with cancer and their caregivers at a hospice in London (UK), showed that averaging preferences at different points in time, 77% of the patients and 59% of the caregivers preferred home [18]. However, 12 other high quality studies were published since 2000, increasing the diversity in populations, study designs, and in the phrasing of the questions used to assess preferences. The findings became more heterogeneous (with the percentage of people who preferred home ranging 5% to 100%). As a result, the strength of evidence decreased from high to moderate.

#### Heterogeneity between and within population groups

**Figure 3:**
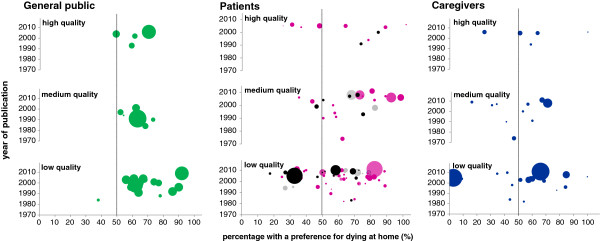
**Preferences for dying at home: population group, study quality, year of publication.** Footnote: The three plots show the percentage of participants expressing a preference for dying at home in each study by population group (general public, patients and caregivers). The number of dots indicates the number of studies included and the area of the dots indicates the number of participants in each study (largest sample size was 4,198 participants). Black and pink dots in studies conducted with patients distinguish between studies where >50% of patients had cancer (pink) and studies where ≥50% had non-malignant diseases (black); grey dots represent studies where the proportion of patients who had cancer was unknown.

The evidence within different population groups largely mirrored the general findings. Plots of percentages amongst the main groups – general public, patients and caregivers – confirmed that >50% or more preferred dying at home across studies in all three groups (Figure 3). However, the plots also revealed wide heterogeneity between studies, particularly amongst those with patients (and to a lesser extent caregivers), when compared with the general public.

Once study quality was taken into account, evidence that the majority prefer dying at home graded moderate with two exceptions (Table 1). The evidence on general public was found to be strong, with three of four high quality studies from Australia and Spain showing that most of the general public preferred home [23,25-27]. The evidence on older people was found to have low strength. There were no high quality studies reporting the percentage of older people who preferred dying at home. A medium quality study in Quebec (Canada) found that 34% of 138 people aged 55 or over recruited via senior organizations preferred home [28]. Eight low quality studies reporting percentages from 39% to 94%, the latter in a small-scale study with 49 people aged 60 or over recruited via newsletters for seniors, also in Canada [29].

Qualitative research filled the gap of high quality quantitative information on older people, with five studies exploring at length the preferences of this group in Canada, Ghana and the UK [29-33]. The findings showed that older people wished they would not have to move from their home. However, there were fears of burdening the family and requiring full-time nursing care due to dependency from a chronic limiting illness or while dying. Normative and moral values (i.e. cultural and religious beliefs involving death at home), and practical issues also played a role for older people (e.g. family witnessing suffering and having to manage pain and the dying body within the domestic space, concerns with the quality of care at home, presence of professionals in the home, involvement of children in intimate care, poor material conditions at home).

The preferences of the three main population groups are now examined in more detail.

#### General public

Twenty-four studies showed that the majority of the general public (52% to 92%) preferred dying at home. This was contradicted by two studies; one recruited 122 participants from local churches and a waiting room in a primary care center (US) of which 38% said they would choose to die at home if they “had a life-threatening illness” [34], and a study (high quality) conducted with 725 attendees in three health centers in Gipuzkoa (Spain), where 49% said that if they had cancer in terminal stage they would prefer to spend the last days at home [27]. Among the four high quality studies, estimates of a home preference ranged from 49% in Spain to 70% in Australia [23,25-27].

Three US studies (two nationwide telephone surveys conducted in the 1990s by market and social research companies for the National Hospice Organization, and one household survey in South Dakota) reported notably high percentages (86%, 90% and 92%) [35-37]. Influenced by these studies, the US findings showed more variation than the eight surveys in Europe, which reported home preferences in Ireland (67%), Italy (62%, 64%), Spain (49%, 59%, 61%) and the UK (56%, 63%). A telephone survey conducted in two counties in Georgia (US) in 1990, found that although 73% preferred to die at home, 70% would like to be cared for in hospital in the prospect of being very sick [38]. There appeared to be no time trends, as shown in Figure 3.

#### Patients and caregivers

Overall, 94 studies determined the prevalence of a preference for dying at home amongst patients (n = 58 studies), their caregivers (n = 2), or both (n = 34). Nearly half were conducted in the UK (n = 29) or the US (n = 17), but 21 other countries were also covered. In 54 studies, >50% of the patients had cancer and in 23 studies the majority had other diseases (in 17 studies the percentage with cancer was unknown). In 82 studies the patients were deemed terminally ill.

Sixty-six studies out of 92 examining patient preferences found that most wished dying at home but there was wide variation (percentages ranging 18% to 100%, Figure 3). Amongst the ten high quality studies and excluding two small-scale outliers in each extreme, estimates of a home preference ranged 31% to 87%. Twenty-six studies from 13 countries found that <50% of patients preferred home – in the UK (7 studies in England and 1 in Scotland), the US (4 studies), Spain (3 studies), Japan (2 studies), South Korea (2 studies), and Australia, Ethiopia, Rwanda, Sweden, Taiwan, Uganda, and Zimbabwe (with 1 study each). The lowest percentage (18%) was found by a Spanish study regarding the preferences for place of death of 102 patients with congestive heart failure or end-stage dementia who died in two acute care hospitals, based on caregiver accounts one month after the patient died [39]. The highest percentages in studies where most patients had non-malignant conditions (89% and 82%) were lower than in studies where most patients had cancer (100% and 92%).

Twenty-two out of 36 studies examining caregiver preferences found that the majority preferred their relatives to be at home, with less variation than in studies with patients (Figure 3). Estimates of a home preference ranged 25% to 64% amongst the five high quality studies, excluding one small-scale study at a community care hospice in Durham (US) where all caregivers preferred the patient to be cared for at home [40]. The lowest estimates were found in medium to low quality studies from Japan (3%, 3% and 15%) and Ethiopia (9%) [41-44].

**Figure 4:**
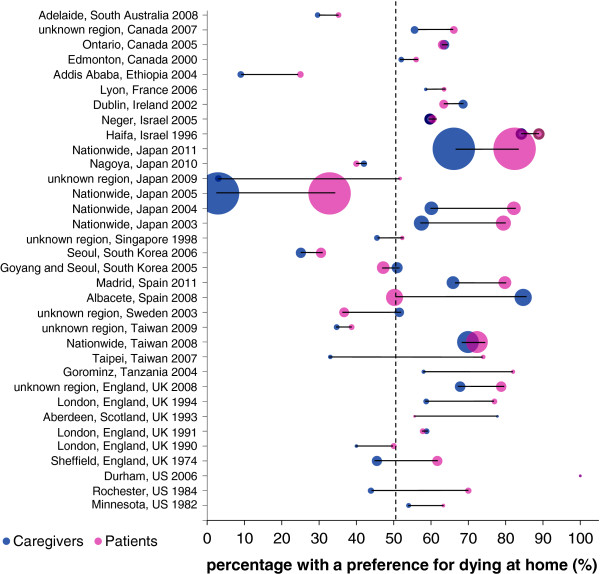
**Preferences for dying at home amongst patients and their caregivers.** Footnote: The plot shows in each study the percentage of patients and their caregivers expressing a preference for dying at home. The number of dots indicates the number of studies included (n = 34) and the area of the dots indicates the number of participants in the study (largest dot represents 4175 participants). Blue dots report to caregivers and pink dots to patients (dots are superimposed for one study as the percentages were 100% for both patients and caregivers). Country region was unknown in six studies.

Comparisons of home preferences for paired groups of patients and caregivers (provided by 34 studies, five of high quality) showed that the differences between the two groups were generally small – 17 studies found differences of <10% (Figure 4). A home preference was more frequent amongst patients than amongst their caregivers in all but eight studies (two of high quality). However, statistical tests were rarely performed. Of the five high quality studies, two tested for differences and reported conflicting findings [45,46]; hence, the evidence on whether patient and caregiver’s preferences for home differ was deemed inconclusive. A telephone survey of 216 bereaved relatives of patients who received home care in Ontario (96% with cancer) [45] showed that relatives were more likely to prefer an institutional death (14% as compared to 5% of patients; p < 0.001). In contrast, a study of 371 cancer patients and 281 caregivers in seven university hospitals and one national cancer centre in South Korea [46] found no differences regarding preferences for a home death (47% patients and 51% caregivers, p = 0.10), although caregivers were less likely to prefer home as place of care compared to patients (49% vs. 53%, respectively; p = 0.02). The largest discrepancy was found in a Japanese study with nursing home residents with middle or advanced stages of dementia and their caregivers, where 15/29 patients (52%) but only 1/31 caregivers (3%) preferred the patient to die at their own home [42].

This heterogeneity between and within groups was corroborated by qualitative research. The case of advanced dementia was examined in focus groups with 39 family members of severely impaired nursing home residents in Minnesota, US [47]. The findings revealed complex negotiations between family and patients about moving into a nursing home (often dictated by need rather than preference); the use of the patient’s personal historical identity and previous wishes to guide decision-making in the absence of overt preferences; and difficulties for the family in balancing the patient and their own wishes. Studies conducted in Canada, Japan and Denmark, mainly about cancer patients, showed that in some situations a preference for home was dyadic and equally important to patients and caregivers [48,49]; the result of a ‘mutual pact’ or promise to the patient, sometimes made in the context of a hospital admission or involving reciprocate care [50,51]. Some caregivers expressed a sense of achievement when the patient died at home, whilst others felt lack of choice, guilt and sorrow, depending on the outcome [50,51]. In Morecambe Bay (UK), Thomas et al. studied the preferences of 41 patients with advanced cancer and found they were strongly influenced by an assessment of caregivers’ capacity to care, irrespective of caregivers’ expressed desire to do it [52]. In Örebro (Sweden), Sahlberg-Blom et al. interviewed bereaved relatives of 56 cancer patients and found that patient participation in end of life decisions, including on where to die, varied from self-determination and co-determination to delegation and non-participation, depending on context, patient’s personality, social network, cultural values and the extent to which their wishes and those of the caregivers could be supported [53].

### Changes over time

Overall, 15 studies (3 high quality) measured if preferences changed over time (one of the general public, six of patients, seven of both patients and caregivers and one including professionals, patients and caregivers). In 13 studies, the data were collected prospectively, either through repeated interviews or observations with participants (n = 8) or from patient records (n = 5). Two studies enquired retrospectively (e.g. “Have your thoughts changed in any way in the last few months/weeks?”). Twelve studies (two of high quality) tracked individual changes (nine studies of patients, two of caregivers and one of both). The evidence was inconclusive as only the study with members of the public reported the statistical significance of the observed changes. A summary of the findings follows.

#### Group changes

An experiment with 183 members of the general public recruited via choir and music associations in the Netherlands, assessed preferences prior and after showing the participants’ stories in text or video reflecting a combination of attributes relevant to place of death, and compared with a direct method of value elicitation [54]. The authors found significant shifts after text and video stories were shown. Participants became more negative about choosing to die at home and more positive about choosing to die in hospice, particularly older participants. Those who watched the video stories also became more positive towards nursing homes.

Townsend et al.’s study, conducted in 1986–87 in a London hospital, was the only study to comment on the significance of changes in patient preference over time [17]. The authors found a decline in home preferences in realistic circumstances (from 58% to 50%) and an increase in ideal circumstances (from 68% to 72%), amongst 84 terminally ill patients. Townsend et al. stated that “the change in preference was not of a significant order”, although it was unclear whether statistical tests were performed. Findings of three other studies may have reached statistical significance if tests were performed, of which two suggested declining preferences for home. A study with 125 older patients in a community-based call program in Baltimore (US) found that 99 patients changed their preferences for place of death, all but one from hospital to home [55]. Changes in the opposite direction were reported amongst home care patients at a London hospice in 1984–86 [18]. Preferences for home care decreased from 100% to 54% amongst 77 patients and from 100% to 45% amongst their caregivers in the last eight weeks before the patients died. A study embedded in the RCT of a palliative care program in Adelaide (Australia) was the most recent study to examine preference change through repeated interviews. This also reported decreases in a home preference, amongst 71 dyads of patients and caregivers [56]. Preferences for home as place of care fell from 87% to 71% amongst patients and 85% to 66% amongst caregivers, whilst a preference for home death fell from 41% to 35% amongst patients and from 42% to 30% amongst caregivers.

#### Individual changes

Changes in preference were documented for 277 of 1,395 patients (20%) across ten studies (two of high quality). However, this ranged from 2% to 80%. UK studies found changes for at least 31 of 243 palliative care patients in Yorkshire (with no evidence that changes were assessed for all) [57], four of 41 terminally ill cancer patients in Morecambe Bay (high quality study) [52], 5 of 21 terminally ill patients in a GP practice in Cambridge [58] approximately 61 of 166 patients seen by a hospital palliative care team in London [59], 5 of 298 patients in three hospices in the south east of England [60], four of 30 terminally ill cancer patients in a general practice in Scotland [61]. In addition, preferences changed for 99 of 125 older patients in the Baltimore community-based house call program (high quality study) [55]; 23 of 71 terminally ill patients in the Adelaide palliative care program [56], eight of 20 terminally ill patients who wanted to die in hospital in Ontario [62] and 37 of 380 cancer patients under the home palliative care team in Madrid [63]. The direction of changes varied but was most commonly from hospital to home, home to hospice and from home to hospital.

Three studies, none of high quality, found that less than a third of caregivers changed preference for the patient’s place of death – 21 of 71 caregivers of patients in the Adelaide palliative care program [56]; 3 of 18 caregivers of cancer patients at a hospital palliative care service in London [64] and 22 out of 205 caregivers (11%) in Grande et al.’s study with patients referred to five Hospice at Home services in Cambridge (UK) [65].

#### Qualitative research

Eight studies conducted in Australia, Canada, Sweden, the UK and the US added qualitative information on changes over time, mostly presented in case stories [40,52,53,66-70]. Different patterns emerged, which resonated with the quantitative findings. Strong and consistent home preferences from patients and caregivers were reported, but so were cases of changes in preferences from home to institutions and from institutions to home. Health professionals in Australia and Canada said that patients and caregivers often change their minds [69,70]. Preferences changed from home to institutions due to uncontrolled pain and other symptoms (“she had terrible time breathing”, incontinence, mental impairment), acute events (e.g. falls, injuries), treatment of reversible conditions for comfort and to maximize length of life, imminent death, caregivers burden/inability to safely care at home, increased need for care and dependency (“as I cannot go to the loo”, “when he discovered he was incapable of being the host in his own home”), “naivety” on what to expect (“I’ve been watching too much TV, if I had known…”), and possible traumatic effects on children. Preferences changed from institutions to home due to the recognition that “prognosis was grim”, limited beds available (“said she couldn’t stay any longer”), unsatisfactory/uncomfortable hospital experiences (e.g. fighting for adequate pain control in hospital), and when assured of support from services at home.

### Differences between place of care and place of death

Four studies (two of high quality) provided inconclusive evidence on whether patient preferences for place of care and for place of death differ. All four found that the majority preferred to be cared for at home and that fewer preferred to die at home, but only one carried out statistical tests. This study involved 380 patients with advanced cancer under a home palliative care team in Madrid (2004–06); 89% preferred to be cared for at home and 80% preferred to die at home (p < 0.001) [63]. Another study of 71 patients embedded in the RCT of the Adelaide palliative care programme (2002–04) found that more than two thirds preferred to be cared for at home (87% when first assessed and 71% when last assessed) but only 40%-35% wanted to die at home [56]. No statistical tests were performed, though, and the same applied to two studies in South Korea, which found differences no greater than 6% [46,71].

Findings on caregivers were equally inconclusive with four studies showing contradictory results and statistical significance untested. The two South Korean studies suggested home was more frequently preferred as the place of death than the place of care amongst caregivers (but with differences no greater than 2%) [46,71], whilst two studies – the Adelaide palliative care program RCT and Hinton’s study of home care patients at a hospice in London (1984–86) – suggested the opposite [18,56].

Qualitative research showed distinctive conceptual features of preferences for place of care and for place of death [40,50,72,73]. A “definite” or “desperate” desire to remain at home as long as possible, with the ultimate hope to die at home or until admission if required, was viewed as dignifying if by the time the move happened the person was not aware of it anymore [50,72]. Perceptions of family’s ability to care impacted on place of care preferences, whereas consequences of witnessing death at home (e.g. for children) impacted on place of death preferences [50,73]. Regardless of place of death, some surviving spouses said the value was frequently on the place where the majority of terminal care was provided and expressed satisfaction at having mastered the time spent at home [40,50].

## Discussion

In this systematic review we found numerous studies providing moderate evidence that the majority of people prefer dying at home (this was reported in 75% of 130 studies). We also found, based on 10 studies, that around four fifths of patients did not change preference as their illness progressed. There was, however, wide variation. This was observed even amongst high quality studies. Heterogeneity was lowest amongst general public studies and greatest amongst patient studies. We also found indications that a preference for home may be less frequent amongst lay caregivers and older people with qualitative research suggesting that although home is generally the ideal preference, circumstances may make this seem impossible. Qualitative research also revealed a conceptual distinction between preferring home as the place of care and as the place of death, although the evidence was inconclusive on whether there are statistically significant differences between the two.

### Majority prefers to be at home

Our findings showed that this applies to different groups in the population – most importantly to patients (in 87% of the studies they were deemed terminally ill). Having found consistent results observed by different researchers in different places with different samples strengthens the likelihood of the finding to be true [74]. Although the body of evidence identified presented important methodological weaknesses, such as lack of clarity regarding the questions used to elicit preferences, there are now more high quality studies than in 2000 [15]. Still, caution is needed due to the observed variation in prevalence estimates of a preference for home, even amongst high quality studies (ranging 31% to 87% amongst patients, excluding outliers). This highlights the need to take the heterogeneity seriously and explore whether it may reflect real diversity in preferences for dying at home.

### Heterogeneity

Our review included a large number of studies with varied features and populations. Methodological differences (e.g. quality of studies) and clinical differences (e.g. population group) explained some of the variation in findings between studies, but not all. There was still considerable heterogeneity within population groups and when ‘controlling’ for quality (as shown in Figure 3). This suggests that although the majority prefers dying at home, there may be diversity in individual preferences. This conclusion, however, takes study-level variation as indicative of individual-level variation and thus carries the risk of an interpretation error (“ecological fallacy”) [75].

Data on patients and caregivers from the same study allowed us to make more direct and robust comparisons. Although statistical significance was rarely tested (hence the evidence was inconclusive), in the majority of the studies a home preference was higher amongst patients than amongst their caregivers. This corroborated previous findings [15]. The difference, should it be statistically significant, is important as the care provided by caregivers and their preferences are strong factors associated with the likelihood of the patients to die at home [21]. Qualitative findings suggested that caregivers commit to providing care and to address the patient’s preference to be at home, to then become aware of the complexities involved. This highlights the importance of good communication of preferences and concerns between patients and caregivers throughout the process, and the need for practical and emotional support to caregivers, to meet the patient’s preference when possible and to minimize the risk of difficult bereavement for caregivers.

### Ideal and realistic preferences

Public preferences for home do not appear to be higher than patients’ and caregivers’ but the evidence is stronger and more homogenous (Figure 3). This finding suggests there may be less consensus in the face of reality and, as Townsend et al. suggested, an important distinction between preferences in ideal and realistic circumstances [17]. Real life situations reported in qualitative research raised the issue of whether it is possible to prepare for acute events and to encourage things that have been found to keep ideal preferences possible (e.g. services at home, available and able caregivers). However, it is still not clear cut whether preferences for patients and caregivers change significantly over time. There is a real need for high quality studies on this matter.

### Limitations

Our review has several limitations: search boundaries, the subjectivity introduced by the quality and grading criteria (although this involved independent reviewers and disagreement checks), and the reliance on data provided by relatively low quality quantitative research (although high and moderate strength evidence was only taken from high and medium quality studies). It is arguable that the quality assessment scales could have placed greater emphasis on how the data were collected and on the measurement of preferences, although this would give greater weight to a specific aspect of quality in detriment of others. Nevertheless, the way preferences are assessed is very important when analysing the findings. We urge consideration of any applicable measurement biases while interpreting the results of the different studies; to help with this we have provided the exact wording of the questions used to elicit preferences when this was known (Additional file 4). The review focused on a preference for home but we acknowledge the importance of other places for care and death, particularly hospices and palliative care units (studies suggest they are the second most frequent preference) [15] and care homes (increasingly important in ageing populations). Furthermore, the searches resulted in a large number of studies related to cancer patients (cancer was the only disease-specific search term used). A review targeted to non-malignant conditions such as COPD and advanced dementia may discover further literature for increasingly relevant groups for end of life care provision.

## Conclusions

In this systematic review, we observed that the majority of people prefer dying at home. This is aligned with the direction taken by current end of life care strategies to target a home setting [11]. Notwithstanding, even in countries where these strategies exist, the majority of people still do not die at home [3,14,76]. This highlights the need for stronger action on factors previously found to influence death at home [21] so that more are able to have their preferences met. There is also the need for further research to understand what factors influence death at home for people dying from non-malignant conditions, where the evidence is thinner and the chances of dying at home are generally lower than for cancer patients [14,76].

At the same time, our findings urge rigorous and regular monitoring of preferences, and consideration of the diversity in views, with attention to older people and caregivers. The strength of the evidence supporting that the majority prefers home to other settings is not as strong as it once was and there is a substantial minority of patients and caregivers for whom home is not the first choice or who change their mind. In face of an increasing demand of end of life care [3,4,8,9], the findings highlight the importance of allowing for a degree of diversity and flexibility in service planning, alongside the focus on home care.

## Abbreviations

AIDS: Acquired immunodeficiency syndrome; COPD: Chronic obstructive pulmonary disease; HIV: Human immunodeficiency virus; MND: Motor neurone disease; RCT: Randomized controlled trial; UK: United Kingdom; US: United States.

## Competing interests

The authors declare that they have no competing interests.

## Authors’ contributions

BG and IJH conceived the idea and obtained funding; BG and NC conducted the searches, identified and retrieved the studies, extracted the data and assessed quality. SH conducted quality assessment for quantitative research and MG for qualitative research. All authors developed the methods, analyzed and interpreted the data, wrote the report and are guarantors. All authors read and approved the final manuscript.

## Pre-publication history

The pre-publication history for this paper can be accessed here:

http://www.biomedcentral.com/1472-684X/12/7/prepub

## Supplementary Material

Additional file 1 PRISMA checklist. Click here for file

Additional file 2 Search strategy. Click here for file

Additional file 3 Quality assessment criteria for quantitative and qualitative research. Click here for file

Additional file 4 Questions used to assess preferences. Click here for file

Additional file 5 High quality studies. Click here for file
