# Supplementary material for: Heterogeneity and changes in preferences for dying at home: a systematic review
Source: BMC Palliat Care. 2013 Feb 15;12:7. doi: 10.1186/1472-684X-12-7 (PMC3623898; doi:10.1186/1472-684X-12-7)
Supplement: Additional file 2 — Search strategy. [file 1472-684X-12-7-S2.doc]

**Additional file 2 –Search Strategy**

Electronic searches in four databases - MEDLINE (1966-2011), EMBASE (1980-2011), psycINFO (1967-2011) and CINAHL (1982-2011) using a combination of MESH headings and 22 keywords.

MESH HEADINGS

MEDLINE

*palliative care*

*hospice care*

*hospices*

*terminal care*

*terminally ill*

EMBASE

*palliative nursing*

*cancer palliative therapy*

*palliative therapy*

*terminal disease*

*terminal care*

*terminally ill patient*

*hospice*

*hospice care*

*hospice patient*

*hospice nursing*

psycINFO

*palliative care*

*hospice*

*terminal cancer*

*terminally ill patients*

*death and dying*

CINAHL

*palliative care*

*hospice and palliative nursing*

*hospice patients*

*hospices*

*hospice care*

*terminal care*

*terminally ill patient.*

KEYWORDS

*palliative*

*hospice**

*terminal care*

*terminally ill*

*terminal illness*

*advanced disease*

*advanced cancer*

*end of life*

*end stage*

*death*

*dying*

AND

*location*

*place*

*setting*

*home*

ADJACENT WITHIN 3 WORDS TO

*care*

*death*

*dying*

*die*

ADJACENT WITHIN 3 WORDS TO

*prefer**

*wish**

*decision**

*choice**
