# Supplementary material for: Heterogeneity and changes in preferences for dying at home: a systematic review
Source: BMC Palliat Care. 2013 Feb 15;12:7. doi: 10.1186/1472-684X-12-7 (PMC3623898; doi:10.1186/1472-684X-12-7)
Supplement: Additional file 3 — Quality assessment criteria for quantitative and qualitative research. [file 1472-684X-12-7-S3.doc]

**Additional file 3 – Quality assessment criteria for quantitative and qualitative research**

**Quantitative research**

Adapted from the NHS Centre of Reviews and Dissemination guidelines for observational studies [19]

| **Items/scoring** | Good  (2pts) | Fair  (1 pt) | Poor  (0 pt) |
| --- | --- | --- | --- |
| 1. Hierarchy study design  (prospective (2 pts), cross-sectional (1 pt), retrospective (0 pt)) |  |  |  |
| 2. Was there a sufficient description of the sample and groups? |  |  |  |
| 3. Were there important confounders and adjustment for these? |  |  |  |
| 4. Is the sample representative and from a relevant population? |  |  |  |
| 5. Are the criteria for inclusion and exclusion explicit? |  |  |  |
| 6. Was the response rate acceptable?  (60% or over (2 pts), between 50 and 59% (1 pt), less than 50% (0 pt)) |  |  |  |
| 7. Were variables reliably and objectively ascertained? |  |  |  |
| 8. Was a rigorous and appropriate analysis used? |  |  |  |

Final score: minimum=0; maximum=16

**Qualitative research**

Adapted from Hawker critical appraisal tool for evaluating qualitative and mixed research [20]

| **Items/scoring** | Good  (3pts) | Fair  (2 pts) | Poor  (1 pt) | Very Poor (0 pt) |
| --- | --- | --- | --- | --- |
| 1. Do the abstract and title provide a clear description of the study? |  |  |  |  |
| 2. Were there a good background and clear aims? |  |  |  |  |
| 3. Is the method appropriate and clearly explained? |  |  |  |  |
| 4. Was the sampling strategy appropriate to the aims? |  |  |  |  |
| 5. Was the analysis sufficiently rigorous? |  |  |  |  |
| 6. Have ethical issues been addressed, and ethical approval gained? Has the relationship researchers- participants been adequately considered? |  |  |  |  |
| 7. Were the researchers reflexive/aware of own bias? |  |  |  |  |
| 8. Is there a clear statement of the findings? |  |  |  |  |
| 9. Are the findings transferable to a wider population? |  |  |  |  |
| 10. How important are the findings for policy & practice? |  |  |  |  |

Final score: minimum=0; maximum=30.

Footnote: Guidelines for scoring are available from the authors.
