# Supplementary material for: Heterogeneity and changes in preferences for dying at home: a systematic review
Source: BMC Palliat Care. 2013 Feb 15;12:7. doi: 10.1186/1472-684X-12-7 (PMC3623898; doi:10.1186/1472-684X-12-7)
Supplement: Additional file 4 — Questions used to assess preferences. [file 1472-684X-12-7-S4.doc]

**Additional file 4 – Studies providing the exact question used for eliciting preferences**

| **Study and year^a^** | **Question** |
| --- | --- |
| *AARP Massachusetts 2005* | "If you were terminally ill and could choose where to die, where would you MOST want to die?" PLUS "Where would you want to receive hospice support?" |
| *AARP North Carolina 2003* | "If you were terminally ill and could choose where to die, where would you MOST want to die?" PLUS "Where would you want to receive hospice support?" |
| *Abarshi et al 2009* | "Where did patient wish to die? (at home or with family/in a care home/in a hospital/in a hospice or palliative care unit)." |
| *Abernethy 2006* | “Questions asked included: “Do you have any plans for your future care?” “Where they content with their place of care at present?” And in the setting patients had clear understanding of their prognosis “And if your illness got worse where would you like to be?” |
| *Addington-Hall et al 1991* | "Was place of death right for the patient? Home was the right place for the patient because the patient wanted to die there; Hospital was the right place for the patient because the patient needed hospital treatment or was too ill to be nursed at home; Hospital was the wrong place for the patient because the patient had wanted to die at home/nurses had been too busy to give the patient care require". AND "Was the place of death right for the carer? Home was the right place because carer had wanted the patient to die at home/carer acceded to the patient's wish to die at home; Hospital was the right place because the patient had required hospital treatment/carer could not cope with care of the patient"; Hospital was the wrong place because carer had wanted patient to die at home/carer knew the patient had wanted to die at home" |
| Alonso-Babarro et al 2011 | Place of care: “Given your health state, where would you choose to be cared for?” Place of death: “When your illness gets worse, where would you rather be?” and “Where would you prefer to die?” |
| *Bajo et al 1990* | “Based on your experience, how many of your terminal patients had wished to die at home? – None of them, 25% of them, 50% of them, 75% of them, all of them.” (Original: Según tu experiencia, ¿que proporción de tus enfermos terminales hubiera deseado morir en casa? – Ninguno, el 25%, el 50%, el 75%, todos.) |
| *Barnato et al 2009* | If that illness got worse, where would you like to spend your last days—in a hospital, a nursing home, or at home? |
| *Beccaro et al 2006* | “Where did the patient die? Did they ever say that there was a place they would like to die? If yes, where was the place? If no, where do you think that they would have wanted to die? |
| *BMJ 2007* | “Where would you prefer to die? “(In a context of having a terminal illness). |
| *Braun et al 2000* | "Where would you want to be if you were dying? (home hospital, nursing home, other)” |
| *Brazil et al 2005* | Preference for place of death was examined using a set of six questions: 1. Where did (he/she) die? (home, hospital, nursing home, other) 2. Did (he/she) ever say that there was a place where (he/ she) would have wanted to die? (yes, no, don't know) 3. Where was this place? (home, hospital, nursing home, other) 4. (Ask if discrepancy between questions 1 and 3) What would have been needed to be different so that could have died at (his/her) preferred place? (please explain) 5. Do you feel that where (he/she) died was the right place for him/her? (yes, no, [please explain], don't know) 6. Did you have a preference for where (he/she) died? (yes [home, hospital, nursing home, other, please explain], no, don't know) |
| *Brown et al 1994* | "Should treatment to cure or contain your illness seem to be failing (or already have failed, in the case of patients with end-stage disease), would you prefer to be cared for at home, as an inpatient at a hospital or as an outpatient?" |
| *Brown et al 1990* | “When you think of yourself/the patient being at home or in the hospital, what do you see as the “good” or “bad” of each? What are the advantages of each?” AND “Can you ever see yourself/patient going to the hospital again (or going back home)? If what would make you want to do that, or consider that? If not, why not?” |
| *Cantwell et al 2000* | "I would like to receive care at home until I die" (patient). "I would like to provide care at home to my family until he/she dies" (caregiver). 7 point Likert scale (from strongly agree to strongly disagree). |
| *Catt et al 2005* | “If I were severely ill with no hope of recovery, I would rather be cared for in a hospice than at home”. “If I were severely ill with no hope of recovery, I would rather be cared for in a hospital than a hospice”. “If I were severely ill with no hope of recovery, I would rather be cared for in a hospital than at home”. These statements were part of a questionnaire to measure attitudes to and preparations for death, and attitudes to and knowledge of hospices and palliative care services. Mean scores obtained, on the attitude scale a score of 3 equals neutral opinion, <3 suggests agreement and >3 suggests disagreement |
| *Charlton et al 1991* | "If you were told you were going to die, which of the following places would you prefer? Home, hospital or hospice? |
| *Chvetzoff et al 2006* | Where would you rather be treated in your terminal period of life, if possible? A) I’d rather be treated in hospital; B) I’d rather be treated at home. *(Original: O*ù *vous parait-il préférable, dans la mesure du possible, d' organiser cette période terminale de la vie? A) Plutôt en hospitalisation, B) Plutôt a domicile)* |
| Crédoc 2003 | “What would be the ideal end-of-life support for people with serious illness or the elderly in terms of: medical care, psychological support, place of care, relationship between relatives, family, patient caregivers and others, place for volunteer support” (*Original: Quelle serait la prise en charge idéale lors de la fin de vie des personnes atteintes de maladies graves ou des personnes âgées?*  *Au niveau des soins médicaux, de l’aide psychologique, de la place de l'entourage, du mode de relation entre les proches, la famille, le malade, les soignants et autres, de la place des bénévoles d'accompagnement?)* |
| *Di Mola et al 2001* | "If you were suffering from an incurable and fatal disease, in which of the following places would you prefer to spend the last days of your life?" |
| *Duffy et al 2006* | 2 questions amongst several from End-of-Life Trade-Off Scenarios. “Scenario: Pretend that you have just been diagnosed with a terminal illness and have been told that you are expected to live only 6 more months. During this time, you will be faced with several difficult choices to make about your care. A series of choices will be presented to you. I would like you to tell me what your choices would be and why”.  “Scenario 1: Dying at Home Versus Being a Burden to Family. You are being discharged from the hospital but require a great deal of personal care. Would you rather go home knowing that your care would be difficult for your family, or would you rather go to a nursing home or hospice?”  “Scenario 5: Intensive Care Even If No Hope of Recovery. You have the chance to stay in intensive care and have everything done to prolong your life as long as possible or go home and spend your remaining days spending time with your family. Which would you rather do?” |
| *Fernandez et al 1996* | "If you had an incurable terminal illness, where would you prefer to be cared for in your final moments or where would you prefer to die?” *(Original: Si usted padeciese una enfermedad incurable en fase terminal, ¿donde preferiría ser atendido en los momentos finales o donde preferiría morir? En su casa, en el hospital, en una residencia (de ancianos, casa de acogida), otros)* |
| *Fernandez Suarez et al 2002* | “Where would you prefer to die? “(In a context of having a terminal illness). *(Original: ¿Donde preferiría morir? Domicilio, hospital, residencia, otros)* |
| *Formiga et al 2004* | "If recovery from your illness was unlikely, what site of death would you prefer: home or hospital?” |
| *Fried et al 1999* | “If you had the same illness that brought you to the hospital again in the future, and you could receive the same treatments at home or in the hospital (for example, intravenous medications, oxygen, blood tests, x-rays), and you would have an equal chance of getting better in the home and in the hospital, and you would be equally safe at either place, where would you prefer to be taken care of? At home you would receive a daily home visit by a nurse and would have a home health aide several hours a day, and this would be paid for by Medicare” (Nonterminal illness). “Now I am going to ask you if the following would change your mind about preferring [the home, the hospital]. If it looked as if you were not going to recover from the illness, regardless of where you were taken care of, would you. . .1. Still prefer [home, hospital] 2. Now prefer instead [hospital, home] (Terminal illness). |
| *Gheorghe et al 2011* | “If you were to develop dementia (in the past called ‘‘senility’’ or ‘‘senile dementia’’) where you lose your ability to care for yourself, could no longer live at home and make your own decisions, would you rather: 1. Go to a nursing home and then if I get sick, don’t send me to the hospital. Just keep me comfortable and let me die with my family at my bedside. 2. Go to a nursing home and if I get sick, send me to the hospital until I get better enough to return to the nursing home. 3. Keep me at home with my family at my bedside in case I get sick.”  “Are there any conditions of severe dementia when you would NOT want to go to hospital but rather be kept comfortable and allowed to die with family at your bedside (circle all that apply): 1. If I could no longer recognize my loved ones, 2. If I could no longer eat because food falls into my lungs, 3. If I could no longer toilet myself, 4. No, I would want to go to hospital and receive treatment no matter what.” |
| *Gilbar et al 1996* | “Where, in your opinion, does the patient wish to be looked after?” |
| *Gyllenhammar et al 2003* | "Had (the next of kin) wished from the beginning that the patient should die at home? If yes, did the next of kin regard the death at hospital acceptable when the illness took a new turn?" (Death in institutional care). “Had (the next of kin) wished from the beginning that the patient should die at home? Had the patient stated that he/she wished to die at home?" (Death at home) |
| *Health Planning Council 2001* | "If you were terminally ill, where do you think you would want to die?" |
| *Higginson 2003* | "Where would you want to be cared for if you were dying? Home, hospice, hospital, nursing home, residential home, other, don’t know” |
| *Hinton 1994* | “"Given how you are now where do you think you would be best cared for?" |
| *Ho et al 2004* | "Once the decision to withdraw therapy is made, and if the patient is expected to survive more than 12 hours after stopping ICU treatment, where would you consider the best place (or preferred place) to offer palliative/comfort care for the terminal critically ill patient if there is no resource constraint?" (number from 1 to 6, one being the commonest place, omit the box if not practised – ICU, High dependency unit, general ward in the same hospital, hospice ward, patient’s home, others – please specify)). |
| *Howell et al 2011* | ‘‘If your condition worsens, what is your hope about where you would like to receive care?’’ If deemed appropriate by health care providers, patients also were asked about preferred place of death. |
| *Hwang et al 2003* | "If you could die at a place of your own choosing, where would it be? In a hospital, at home, not sure" |
| *Iecovich et al 2009* | "If you know that you are going to die and can receive treatment in any place, where would you like to spend your last days? (a) at home, (b) in a hospital, (c) in a nursing home, (d) in a hospice, (e) elsewhere, or (f ) don’t know.” |
| *Ingleton et al 2004* | "Did they say where they would like to die (yes, no, don’t know)? If yes, where was that place? (at home, community hospital, old people’s or nursing home, hospice, general hospital, other)”. “Do you feel that they died in the right place (Yes, it was the right place; no it wasn’t the right place, not sure)?” and “Why was it not the right place (It wasn’t where they wanted to die; care they received there was poor; too far away from family and friends; other)?” |
| *Irish Hospice Foundation 2004* | "Where would you want to be cared for if you were dying? (In a hospital, in your own home, in a hospice, in a nursing or residential home, other, don’t know)" |
| *Koffman et al 2004* | “Did the deceased ever say that there was a place where (he/she) would have wanted to die? If yes, where was the place? (at home, in a hospice, in a hospital, or somewhere else). What about his/her caregivers or family – did you or they have enough choice about this? How do you feel about he/she dying at home rather than in hospital (or in hospice/hospital/institution rather than at home)? |
| *Kwon et al 2009* | “ Ideal place of death is __________ (Home/Hospice/Hospital/Nursing home/Other)” |
| *Lee at al 1998* | "Many people insisted on being discharged home when they knew that time was short. Some were admitted into hospital when they knew that their condition was deteriorating. Where would you like to be if that happened?" "If your symptoms were controlled, would you like to be cared for at home till you die?" "If we could provide support for you at home in the form of visits from nurses and doctors, would you like to be at home?" |
| *Martineau et al 2003* | 22 items; a 5-point scale associated with each question. The global scores associated with the various constructs could vary between 1 (absolute choice in favour of the hospital) and 5 (absolute choice in favour of the home). Intention component: “I intend to choose the home as place of death, like my close friend” and “I intend to choose the hospital as place of death, unlike my close friend.” A 5-point scale ranging from highly improbable to highly probable measured these items. The second pair of questions was posed as follows: “What are the chances of you deciding to choose the home as place of death, like your close friend?” and “What are the chances of you deciding to choose the hospital as place of death, unlike your close friend?” For these two items, the response scale ranged from 0% chance to 100% chance. Cognitive component: “The fact of choosing the home as place of death, like your close friend, would be in your opinion . . .” and “The fact of choosing the hospital as place of death, unlike your close friend, would be in your opinion. . . .” The two 5-point response scales were rated for the adjectives highly unreasonable to highly reasonable and highly inappropriate to highly appropriate. Affective component: “For you, the fact of opting for the home (hospital) as place of death, like (unlike) your close friend would be: highly uncomfortable to highly comfortable; highly distressing to highly satisfying; and highly discouraging to highly encouraging. Normative beliefs: “In your opinion, would most of the people close to you approve or disapprove of you choosing the home as a place of death, like your close friend?” and “In your opinion, would most of the people close to you approve or disapprove of you choosing the hospital as a place of death, unlike your close friend?” The response scales ranged from would strongly disapprove to would strongly approve. Social role construct: “Given my family’s habits, I would choose the home as a place of death, like my close friend” and “Given my family’s habits, I would choose the hospital as place of death, unlike my close friend.” “I think that a person suffering from cancer should die at home, as does my close friend” and “I think that a person suffering from cancer should die in hospital, unlike my close friend.” The response scale varied from strongly disagree to strongly agree. Moral norm construct: “If I chose the home as place of death, like my close friend, I would feel irresponsible” and “If I chose the hospital as place of death, unlike my close friend, I would feel irresponsible.” The answers were rated on a 5-point scale ranging from disagree strongly to agree strongly. |
| *McCall et al 2005* | "If everything were possible, where would you choose to be cared for in the end stage of your illness? |
| *Hays et al 2001* | “Where would you prefer to die – hospital, your apartment or cottage, healthcare facility on the CCRC campus, no preference, or do not know?” Also asked: “If you have a preference, how certain is it – unconditional (no change under any circumstance) or conditional on (check all that apply): symptom control; amount, type, or duration of care needed; concerns about family or costs, and “other” concerns?” |
| *McCormick et al 1991* | "If it were necessary, would a stay in a long-term care AIDS facility be acceptable for you after your stay in hospital, relative to a longer stay in hospital? Would you prefer home care to ongoing care in a long-term care AIDS facility or hospital, if it could be arranged?” |
| *McCusker 1984 (two studies in same paper)* | "It is often better for sick people to be treated at home rather than in the hospital". "When the time comes, I would prefer to die in my own home". "The best place for a sick person is in the hospital”. "It is hard for doctors to give good quality care in the home". Scale and end questions. ALSO: “Did you feel at any time that you would have preferred your relative to have been treated at a hospital or nursing home instead of at home? Where there any times that you would have preferred him/her to have been treated at home instead of a hospital or nursing home? Would you have preferred your relative had died at home/in the hospital? In your opinion, would your relative have preferred to die at home/in the hospital?” |
| *McQuillan 2007* | “What about dying at home, in the hospital? If a person has incurable cancer and will soon die is it better that they die at home, in hospital, in hospice, other place? Are there places which are particularly unsuitable, why?” |
| *Merriman et al 1987* | “Would you like to die at home?” |
| *Missoulian Demonstration Project 2000* | "If you were terminally ill, where do you think you would want to die?" |
| *Nagamine et al 1988* | “If you had a serious illness from which you would probably not recover, would you prefer to be hospitalised or remain at home?” |
| *Nebraska Coalition 2007* | "If you could choose where to die... How afraid if at all are you of dying in an institution hospital or nursing home? How important would being able to stay in your home?” |
| *Neubauer et al 1990* | "When the time comes, I think I would rather die in my own home rather than in a hospital" and "If I was very sick, I would rather be in a hospital than to possibly be a burden to my family at home" (4 item Likert scale from agree strongly to disagree strongly) |
| *Pategay et al 2005* | "Where do you want to die?" *(Original: O*ù *souhaiteriez-vous mourir? Domicile, Hôpital, Clinique de gériatrie, EMS, Autres)* |
| *Pritchard et al 1998* | "To what extent do you agree with the statement: If the doctors ever told me that I probably had little time to live, then I would rather go home than stay in the hospital. (1) for not at all, (2) for a little bit), (3) for moderately, (4) for quite a bit, (5) for very much |
| *Ramon et al 2006* | Had/he/she preferred to die elsewhere? Plus preferred place of death (O*riginal: ¿Hubiera preferido otro lugar para morir? No sabe, no contesta. No. Si. AND Lugar preferido para morir. Hogar. Otros.)* |
| *Rosenfeld et al 2007* | "My preference is to spend last days in my own home" - participants asked to confirm if they agreed or disagreed with statement |
| *Arribas et al 2004* | “If you had cancer in terminal stage where would you prefer to spend the last days?” *(Original: ¿Hubiera preferido otro lugar para morir? No sabe, no contesta. No. Si. AND Lugar preferido para morir. Hogar. Otros.)* |
| *Singer et al 2004* | “In retrospect, do you think you would repeat the treatment setting in regard to: The healthcare team treatment (yes or no), family support (yes or no), support from friends (yes or no)?” “Do you think it would have been better for the patient to die at home (yes or no)?”, “Do you think it would have been better for you (the caregiver) for the patient to die at home (yes or no)?” Questions asked for primary caregivers receiving homecare program and caregivers receiving non-homecare programs. |
| *Sives et al 2007* | “What is your opinion on the following statement: 65% of cancer patients would like to die at home – Strongly agree, agree, neutral, disagree, strongly disagree” |
| *Smith et al 1984* | Participants asked to respond in terms of what they would want “as a patient dying of a terminal illness in a hospital”. “If you had a life threatening illness, where would you choose to die? (at home, in a hospital, in a hospice facility, other)” |
| *Stajduhar 2008* | “Assume you could achieve the same high level of quality care at home as well as in the hospital. If your current condition deteriorated and you had only a few days or weeks to live, where would you prefer to die? (patient)”. “Assume you could achieve good quality care at home as well as in hospital. If your family member’s condition deteriorated until the point where s/he was dying, where would you like him/her to be cared for? (caregivers)”. Response categories for both patient and caregivers included “home”, “hospital” and “does not matter”. |
| *Steinberg et al 1997* | "To what extent do you agree with the following statements: If I had a terminal illness I would prefer to die... 5 point Likert Scale for every option - from strongly agree to strongly disagree (GPs and community members). Answer options were: at home, in a hospital, in a hospice, in a nursing home. |
| *Schwartz et al 2003* | Statement “That the person was able to remain at home” as part of 17 distinct characteristics to a concept of good death, respondents indicated the importance of the statement – from 1 (not necessary) to 4 (essential) |
| *Tang et al 2004* | Place of end of life care: “Where would you prefer to receive your future care?” and “Where would you like to spend the last week and days of your life?” Realistic preferred place of death: “Some people prefer to die at home, whereas some people prefer to die in a hospital, an inpatient hospice or a nursing home. Given your present state, if you died today, where would you prefer to be?” Ideal preferred place of death: “Some people prefer to die at home, whereas some people prefer to die in a hospital, an inpatient hospice or a nursing home. If everything were possible, where would you prefer to be?” |
| *Toscani et al 1991* | "If you were suffering from an incurable and painful disease, where would you like to spend the last days of your life? (At home, at the hospital, I’d rather not think about it, it makes no difference to me, Don’t know/can’t answer" |
| *Townsend et al 1990* | "Do you have any plans for your future care?" "What would you like for your future care were it possible?" and also if they were content in their present place of care. If a clear answer was not forthcoming a range of possible alternatives was given, including "Would you like to go to hospital; go home; go to a friend, or stay as at present?" If these questions did not elicit the preferred place of death, and the patient had already acknowledged the diagnosis and prognosis, further questions were put such as, "And if your illness gets worse, where would you like to be?" |
| *Uwimana 2008* | “Where do you prefer to be looked after in terminally ill phase?” |
| *Vig et al 2002* | “When thinking ahead to your own dying, under what circumstances would you prefer to die at home or in the hospital? Why?” |
| *Ward et al 1974* | “Would you have liked to get him/her into hospital (carers)?" "Would you have preferred the patient to have been nursed in hospital (physicians)?" - Question not stated for those who died in hospital. |
| *Webb 2009* | “Opinion about having a loved one die at home was assessed via agreement, disagreement, or neither agreement nor disagreement (i.e., neutral) with seven items relating to the question, ‘‘How would you feel about having a loved one die at home?’’ Responses from these items (e.g., ‘‘I think it is best for a person to die at home,’’ ‘‘I would be comfortable having a loved one die in my home’’) were assigned a score of 1, 0, or 1. Reverse-phrased items (e.g. ‘‘I think my loved one would receive better care in a hospital rather than at home’’) were utilized to reduce response bias and were scored such that responses suggesting a positive view of home death received a positive score” |
| *Wilson et al 2000* | “What do you think is the best place and type of care for senior citizens during the last days of their life?” |
| *World Health Organization 2004 (4 studies)* | Urban residents: “Where do you prefer to be looked after during your illness? (please circle one). Here at home. In hospital. I really do not mind where. Give reasons for your answer.” For family caregivers: “Given a choice, where would you prefer to look after this patient? (please circle one) Here at the patient’s home. In hospital. I really do not mind either way In my own home in the village. Please give reasons for your answer.” Heads of Households or Caregivers of Ill Rural Patients: “Where did the person prefer to be looked after during his/her illness? (please circle one). Here at home. In Kampala In Hoima. In hospital. He was too sick to care I did not know”. Where a terminally ill person is present at the time of the study. “Where does this person prefer to be looked after? (please circle one). Here at his home. In hospital. In his town home where services are available. He is too sick to mind. I do not know.” Where there has been no death in the last 12 months and there is a no terminally ill person present at the time of the study. “If you had a family member with HIV/AIDS or cancer, where would you prefer to look after this person? (please circle one). Here at home. In hospital. I would not mind where so long as the sick person gets treatment.” |
| *Zusman et al 1984* | “If you had a serious illness from which you would probably not recover, would you prefer to be hospitalised or remain at home?” |

**^a^** Full reference available from authors
