# Supplementary material for: Heterogeneity and changes in preferences for dying at home: a systematic review
Source: BMC Palliat Care. 2013 Feb 15;12:7. doi: 10.1186/1472-684X-12-7 (PMC3623898; doi:10.1186/1472-684X-12-7)
Supplement: Additional file 5 — High quality studies. [file 1472-684X-12-7-S5.doc]

**Additional file 5 – High quality studies**

**High quality studies using quantitative methods ^a^**

| *Authors, publication year, aim* | *Population (whom preferences refer to), region and country* | *Design, setting, data time period, response rate (RR), assessment method* | *Results* | *Comments* |
| --- | --- | --- | --- | --- |
| Foreman et al 2004 [25]  To understand the factors shaping preferences and decision-making about settings of end-of-life care | General public  n=2,652  South Australia (nationwide) | Design: cross-sectional population-based survey, through the Health Omnibus Survey, clustered multi-stage sample  Setting: nationwide, households.  Data time period: 2004  RR: 65.9%  Assessment: face-to-face interview at home by trained professionals, participants asked where they would like to die if they had a terminal disease, such as cancer or emphysema. Actual question not provided. | Home preference: **70% would like to die at home**, 19% hospital, 9.8% hospice, 0.8% nursing home (excluding 8% no preference, 4% uncommon preferences). Preferences weighted to age-sex distribution of south Australian cancer deaths 2000-2002 – 58% preference for home.  Factors associated with home preference: (multivariate): younger age, male, place of birth in UK/Ireland or Italy/Greece, better SF36 physical health, poorer SF36 mental health, concern home death would burden family, other concerns about home death, not aware of living wills/ advanced directives/use of medical agents/power of attorney. Preferences similar across areas, except Lincoln (not tested). *No effect:* period of residence in Australia, urban/rural, employment status, marital status, education, interpretation of dying with dignity, appointment of guardian or medical agent, or existence of advanced directive.  Reasons for home preference: Not stated.  Changes over time: not stated. | Very good response rate and robust analysis of factors shaping preferences.  Additional results on factors associated with preferences for hospital, hospice, nursing home. |
| Arribas et al 2004 [27]  To know people’s preferences for information and other attitudes before a cancer diagnosis | General public  n=725  Gipuzkoa  Spain | Design: cross-sectional service-based questionnaire survey with quasi-random sample    Setting: waiting rooms of 3 health centres (one rural, two urban).  Data time period: 2002  RR: 100%  Assessment: 8-item questionnaire administered in the waiting rooms. Question: *“If you had cancer in terminal stage where would you prefer to spend the last days?* | Home preference: **49.3% preferred do stay at home**, 51.0% preferred their family members to stay at home.    Factors associated with home preference: (bivariate): young age, urban. *No effect:* family member with cancer in last 5 years.  Reasons for home preference: Not stated. |  |
| Fernandez-Suarez et al 2002 [26]  To calculate the prevalence of users who want to know their diagnosis of terminal illness and to analyse factors determining their decision | General public  n=388  Asturias  Spain | Design: cross-sectional service-based survey with stratified random sample aged over 17 yrs.  Setting: from lists of health services in six districts of on-demand users  Data time period: 2000-2001  RR: 99%  Assessment: face-to-face interview at home using questionnaire developed by the researchers, Question: “*Where would you prefer to die? “(*In a context of having a terminal illness). | Home preference: **60.7% preferred to die at home** and 22.1% in hospital.  Factors associated with home preference: stated.  Reasons for home preference: Not stated.  Changes over time: not stated. |  |
| Ashby et al 1993 [23]  To provide evidence on community attitudes to certain death and dying issues for a state parliamentary committee on law and practice relating to death and dying | General public  n=462  Adelaide and  three rural areas  South Australia | Design: cross-sectional population-based survey, through a market and social research company, two-stage sample of households and residents  Setting: Adelaide and three major rural areas  Data time period: 1991  RR: 74%  Assessment: face-to-face interview at home, participants asked where they would prefer to die if they were terminally ill. Actual question not provided. | Home preference: **59.3% planned to die at home**, 20.9% in hospital, 5.4% in hospice, 2.5% in a nursing home, 6.2% at another place, 7.8% did not know.  Factors associated with home preference (bivariate): younger age.  Reasons for home preference: Not stated.  Changes over time: not stated. | Safeguards in place to ensure high standards in data sampling and collection. |
| Choi et al 2005 [46]  To examine preferences of patient with cancer and their caregivers for place of terminal care and place of death and the reasons for preferences | Cancer patients and caregivers  n=652  Patients  n= 371  Caregivers  n= 281    Seoul  South Korea | Design: cross-sectional service-based survey.  Setting: waiting room in surgical, medical or radio-oncology clinics in seven university hospitals and national cancer centre.  Data time period: Not provided  RR: 86%  Assessment: self-filled questionnaire (based on previous questionnaires on preferences), including “a series of questions” on preferences for both place of terminal care and place of death and reasons. Patients with caregivers interviewed separately. Actual questions not provided. | Home preference: **53.4% of patients and 49.1% of caregivers preferred care at home; 47.2% of patients and 50.9% caregivers preferred death at home.**  Factors associated with home preference (multivariate): *home care* (patient group): carer as spouse or parent/sibling; (family group) carer as spouse, higher income. *home death* (patient group): male, carer parent/sibling, financial help from spouse; (family group): aged less than 60 yrs, rural environment, carer as spouse. *No effect:* education, religion, time since diagnosis, stage of illness, functional status (ECOG).  Reasons for home preference (most frequent): *home care*: remember personal accomplishments and close one’s life and be with family; *home death*: be with family at death, personal dignity at death and appropriate treatment and peaceful death.  Changes over time: not stated. | Patient preference for home care preference higher than preference for death at home (inverse for caregiver).  Patient and caregiver groups unmatched, direct comparisons between groups not possible.  Additional results on reasons associated with preference for hospital. |
| Yun et al 2006 [71]  To examine personal and situational factors influencing the disparity between patients’ preferences and caregivers for futile aggressive care | Terminally ill cancer patients and caregivers  n=488 (244 each)  Region not provided  South Korea | Design: Cross-sectional service-based survey with convenience sample identified by physicians.    Setting: three university hospitals.  Data time period: 2003  RR: 87%  Assessment: interviews measuring aggressive treatment preferences (patient and caregivers separately), including for end of life care issues such as place of care and death for both patients and caregivers. Actual question not provided. | Home preference: **31.2% patients and 23.1% caregivers preferred home care. 30.5% patients and 25.1% caregivers preferred home death.**  Factors associated with home preference not stated.  Reasons for home preference: Not stated.  Changes over time: not stated. | 72% of patients were hospitalized. Question in context of aggressive treatment preferences might have biased results towards hospital.  Additional results on factors associated with preference for institutions. |
| Tang et al 2004^b^  To investigate the determinants of dying in an inpatient hospice | Terminally ill cancer patients n=180  Connecticut  US | Design: prospective service-based cohort study with convenience sample of patients referred to hospital or home care teams.  Setting: six sites, four tertiary hospitals and two home care teams in New Haven and Hartford counties  Data time period: 2001-2002  RR: 87%    Assessment: structured face-to-face interviews conducted at one point in time before death. Question:  *“Some people prefer to die at home, whereas some people prefer to die in hospital, an inpatient hospice or a nursing home. Given your present state, if you died today, where would you prefer to be?”* | Home preference: **87.4% preferred to die at home**.    Factors associated with home preference (multivariate): no effect of home as preferred place of care on hospice home care use.  Reasons for home preference: Not stated.  Changes over time: not stated. | Additional results on factors associated with preferences for hospice, place of death (23.7% died at home, 29.9% in preferred place with kappa=0.11), factors associated with preferences not met and importance of dying n preferred place. |
| Leff et al 2000 [55]  To determine the prevalence, effectiveness and predictors of planning place of death among older home-bound persons followed by community physician-led call program | Terminally ill patients, caregivers and physicians  n=375 (125 each)  Baltimore  US | Design: analysis of records (data prospectively collected) of patients who died under a house call program.  Setting: community physician-led call program in SE Baltimore.  Data time period: 1995-1998  RR: 80%  Assessment*:* plan of place of death recorded as note in records describing conversation between physician and patient and/or caregiver at time when patient was seriously ill or recovering from episode of serious illness (professional assessment). Actual question not provided. | Home preference: **82.5% planned to die at home**, 16.3% in hospital and one patient in hospice.  Factors associated with home preference not stated.  Reasons for home preference: Not stated.  Changes over time: 100/125 (80%) of patients changed preferences (all cases except one from hospital to home). | Data extraction inter-rater reliability assessed at baseline and improved from 82% to 100%.  Additional results included description of decision-making process, place of death (60.8% died at home, and factors associated with having a plan for care. |
| Hinton 1994 [18]  To assess whether patients with terminal cancer and their caregivers find that competent home care sufficiently maintains comfort and help adjustment | Terminally ill cancer patients and caregivers  n=154 (77 each)  London  UK | Design: Prospective, longitudinal service-based survey with 1:3 random sample of home care patients and their caregivers.    Setting: St Christopher’s hospice home care service. Data time period: 1984-1986  RR: 100%  Assessment: semi-structured informal interviews at home (patient and caregivers separately) each week for eight weeks and, if surviving, fortnightly until six months and then monthly. Caregivers were also interviewed after the patient’s death and asked what care they preferred and where they wished the patient had died. Question for preferred place of care"*Given how you are now where do you think you would be best cared for?"* | Home preference: **Preferences for home amongst patients 54% at last interview and amongst relatives 45%.**  Factors associated with home preference not stated.  Reasons for home preference: Not stated.  Changes over time**:** Preferences for home amongst patients fell from 100% to 54% at last interview and amongst relatives from 100% to 45%. At the follow up, 94% of caregivers preferred home care and only 31% wished the patient died at home (66% preferred hospice). |  |

**^a^** Studies were high quality if they were scored ≥70% in the quality assessment scale.

**^b^** Full reference available from authors

**High quality studies using qualitative methods ^a^**

| *Authors, publication year, aim* | *Population (whom preferences refer to), region and country* | *Design, setting, data time period, assessment method* | *Results* | *Comments* |
| --- | --- | --- | --- | --- |
| Evans et al 2006 [40]  To describe caregivers’ reasons for transfer from home hospice to inpatient facilities, preferences for place of care and death, and their experiences during these transfers. | Terminally ill patients and caregivers  n=36 (18 each)  Durham, North Carolina  US | Design: Analysis using grounded theory approach of retrospective interviews with caregivers of home hospice patients transferred to a facility at some point during hospice care. Use of decision rules and independent coding by two reviewers, disagreements solved by consensus.  Setting: university home hospice service.  Data time period: 2003-2004  Assessment: semi-structured home interviews. Caregivers asked to describe story of transfer and open-ended questions including their and patient preference for place of care and death and whether they and the patient would have preferred care at home. Actual question not provided. | Home preference: **all** **had strong preference for home care**.  Factors associated with home preference: not stated.  Reasons for home preference: Not stated. Description of triggers for changes towards institutional preference: patient comfort through treatment of reversible situations, avoid home where too many reminders of life for patient, avoid traumatic experience to child of parent death at home.  Changes over time: due to situation leading to transfer, caregivers and patients did not feel anymore care should be at home (uncontrolled symptoms, acute medical events, patient falls, and imminent death). In light of changing circumstances, it was felt there was no reasonable alternative. Re-prioritizing: treatment of reversible situations for comfort became more important than stay at home at transfer. | Small sample referring to a selected population in home care that experienced transfer (excluded those that did not who may have different views). |
| McCall & Rice 2005 ^b^  To explore what factors influence decisions around place of care | Terminally ill cancer patients n=8  West Highlands Scotland UK | Design: Cross-sectional study, analysis of interviews with a purposive sample of cancer patients in last days or weeks of life identified through general practices, validation against field notes.    Setting: general practices in rural area.  Data time period: Not provided.  Assessment: semi-structured interviews by researcher and clinical specialist nurse in care. Schedule validated by panel of experts.  Questions: *“If everything is possible, where would you choose to be cared for in the end stage of your illness? Why would you choose this place? Have you been able to share these thoughts with your family/carer? Had you given this subject any thought before becoming unwell? Have your thoughts changed in any way in the last few months/weeks? How important is for you to be cared for in the place of your choice in the end stage of your illness (very important to not important at all)?”* | Home preference: **Two patients preferred to be cared for at home,** four patients thought it was impossible to be at home and chose hospital; two had no preference but doubted home was realistic.  Factors associated with home preference: not stated.  Reasons for home preference: Not stated.  Changes over time: All presumed would be cared for at home but reframed with increasing illness and dependency (more important than pain or distressing symptoms). “Better be where could be looked after”. Preference rarely a definitive feeling, events might change and influence choice. Patients wanted to make decisions alone, due to burden over family and none consider how families might feel. Unawareness of possibility of choice and relief experienced by discussing.  Factors associated with home preference: family resource and ability (pressure of care) decisive factor for all 8 patients, no apparent gender differences. Large family not being able to be in hospital with patient. Limited and variable home care. | Small sample size and involvement of researcher in care (acknowledged and discussed). Interesting questions used to assess preferences.  Factors associated with hospital preference included threatened loss of personal dignity and parental role, personal hygiene in front of family, negative experiences with death at home, burden of constant care over long period of time, traumatic experience and difficulty living in house after death. |

**^a^** Studies were high quality if they were scored ≥70% in the quality assessment scale.

**^b^** Full reference available from authors

**High quality studies using mixed methods ^a^**

| *Authors, publication year, aim* | *Population (whom preferences refer to), region and country, dominant method* | *Design, setting, data time period, response rate (RR), assessment method* | *Results* | *Comments* |
| --- | --- | --- | --- | --- |
| Brazil et al 2005 [45]  To determine caregivers perceptions about place of care and place of death and to identify variables associated with a home death | Caregivers of terminally ill patients  (n=216)  Ontario  Canada  Quantitative method was dominant | Design: retrospective service-based telephone survey embedded in bigger study with a consecutive sample of caregivers of patients who died identified through home care services. Answers to two open-ended questions were analysed using content analysis (independent coding by two reviewers, disagreements solved by consensus).    Setting: publicly funded home care services in both urban and rural areas.  Data time period: 2000-2002  RR: 70% (main study had RR 82%).  Assessment: telephone interviews by researchers using a CATI system for recording. Questions: *“Did he/she ever say that there was a place where (he/she) would have wanted to die? Where was that place? What would have been needed to be different so that he/she could have died at his/her preferred place (open-ended)? Do you feel that where he/she died was the right place (open-ended)? Did you have a preference for where he/she died?”* | Home preference: **63.1% of patients (as reported by caregivers) and 63.6% of caregivers preferred home (but difference not significant, only for hospital – 4.7% vs. 13.6%).**  Factors associated with home preference: not stated.  Reasons for home preference: Not stated.  Changes over time**:** Not stated. | Almost all caregivers (93%) reported in retrospect that death took place in the right place.  Additional results included place of death (56% died at home) and dying in patient preferred place (30% did not die in their preferred place).  Content analyses revealed that familiarity and supportive environment was important and that 24-hour nursing care and effective pain and symptom management were perceived as things needed for patients to die at home. |
| McCormick et al 1991 [24]    To determine preferences for various post-discharge long-term care settings recommended by primary care providers and the impact of these views on the resulting discharge dispositions | Patients with AIDS  n=120  Seattle  US  Quantitative method was dominant | Design: cross-sectional service-based survey with a consecutive sample of hospitalised patients with AIDS identified through hospital services. Answers to unstructured part of interview were analysed using content analysis.    Setting: five large hospitals in Seattle providing care for persons with AIDS in Seattle and in Washington.  Data time period: 1989  RR: 70%  Assessment: Near the time of enrolment, patients were asked for their preferences for place of care and aspects of care important for patients, the latter using an unstructured interview. Questions: *“If it were necessary, would a stay in a long-term care AIDS facility be acceptable for you after your stay in hospital, relative to a longer stay in hospital? Would you prefer home care to ongoing care in a long-term care AIDS facility or hospital, if it could be arranged?”* | Home preference: **73% preferred home care if this could be arranged**. 58% found long-term care facilities acceptable as substitute for hospital care.  Factors associated with home preference: not stated.  Reasons for home preference: Not stated.  Changes over time**:** Not stated. | Content analysis revealed that availability of support from loved ones and medical professionals, privacy, a home-like environment, camaraderie, independence and access to medical care were important factors. |
| Thomas et al 2004 [52]  To examine place of death preferences | Cancer patients and caregivers  n=59 (41 patients and 18 caregivers)  Patients  n= 41  Caregivers  n= 18  Morecombe Bay  UK  Qualitative method was dominant | Design: Prospective, longitudinal study using an approximation to grounded theory to analyse interviews with terminally ill patients and caregivers identified by specialist palliative care professionals (mainly community nurses). Use of second reviewer for a sample of transcripts and attention to typical and atypical cases.  Setting: area in North West of England with relatively low percentage of home deaths (22%).  Data time period: 2002-2003  RR: 57%.  Assessment: two face-to-face conversational interviews (all but two at home) separated by four weeks and then telephone tracking up to death (only 17 patients completed up to death). 18 caregivers interviewed after enrolment and nine 12 weeks after the patient died. Preferences for place of death were elicited through sensitive questioning on related issues, more or less directly depending on ease displayed by patients and caregivers (separately as possible). Actual question not provided. | Home preference: **10 patients preferred to die at home** and 9 preferred either home or hospice.  Factors associated with home preference (for both patients and caregivers):  1. Informal care support: patient’s social network and living arrangements, patient’s assessment of caregiver’s capacity to care, patient concern for the welfare of the caregiver/family, caregiver’s attitudes and willingness to care.  2. Management of the body: symptom management, patient’s fears of loss of dignity.  3. Experience of services: patient and caregiver perceptions of the reliability of services and the degree of “safety” they offer, patient’s attitude to hospitals, hospices and nursing homes, patient’s experience of hospitals, patient’s knowledge and experience of community services, patient’s attitude to, and outlook on, death and dying, including religious faith, previous personal experience of death and dying.  Reasons for home preference: Not stated.  Changes over time: only four patients altered (as opposed to completely changing) their preferences. Two that favoured home became more oriented towards hospice, one leaned from “hospice if I am too bad” towards home, and one expanded his preference from hospice to hospice or local hospital. | Qualitative data suggested that preferences are “pure types” and rarely categorized, but took the form of a stronger or weaker leaning in one direction, qualified by speculation of how things might change with events and framed always in contextual factors. |

**^a^** Studies were high quality if they were scored ≥70% in the quality assessment scale.

**^b^** Full reference available from authors
